# Inhibition of EGFR or IGF-1R signaling enhances radiation response in head and neck cancer models but concurrent inhibition has no added benefit

**DOI:** 10.1002/cam4.345

**Published:** 2014-10-30

**Authors:** Uma Raju, David P Molkentine, David R Valdecanas, Amit Deorukhkar, Kathryn A Mason, Thomas A Buchholz, Raymond E Meyn, Kie-Kian Ang, Heath Skinner

**Affiliations:** 1 Department of Experimental Radiation Oncology, The University of Texas MD Anderson Cancer CenterHouston, Texas; 2 Department of Radiation Oncology, The University of Texas MD Anderson Cancer CenterHouston, Texas

**Keywords:** Head and neck squamous cell carcinoma, IGF-1R and EGFR signaling, radiosensitivity, tumor response to radiation

## Abstract

Interaction between the epidermal growth factor receptor (EGFR) and the insulin-like growth factor receptor (IGF-1R) has been well established in many cancer types. We investigated the effects of cetuximab (EGFR antibody) and IMC-A12 (IGF-1R antibody) on the response of head and neck squamous cell carcinoma (HNSCC) to radiation therapy (RT). The effects of cetuximab and IMC-A12 on cell viability and radiosensitivity were determined by clonogenic cell survival assay. Formation of nuclear *γ*-H2AX and 53BP1 foci was monitored by immunofluorescence. Alterations in target signaling were analyzed by Western blots. In vivo tumor growth delay assay was performed to determine the efficacy of triple therapy with IMC-A12, cetuximab, and RT. In vitro data showed that cetuximab differentially affected the survival and the radiosensitivity of HNSCC cells. Cetuximab suppressed DNA repair that was evident by the prolonged presence of nuclear *γ*-H2AX and 53BP1 foci. IMC-A12 did not have any effect on the cell survival. However, it increased the radiosensitivity of one of the cell lines. EGFR inhibition increased IGF-1R expression levels and also the association between EGFR and IGF-1R. Addition of IMC-A12 to cetuximab did not increase the radiosensitivity of these cells. Tumor xenografts exhibited enhanced response to RT in the presence of either cetuximab or IMC-A12. Concurrent treatment regimen failed to further enhance the tumor response to cetuximab and/or RT. Taken together our data suggest that concomitant inhibition of both EGFR and IGF-1R pathways did not yield additional therapeutic benefit in overcoming resistance to RT.

## Introduction

The combination of radiation with chemotherapy, particularly when given concurrently, has improved local-regional (LR) control and overall survival (OS) in patients with locally advanced cancers of several primary sites 1,2. However, the acute side effects and long-term toxicity of chemo-radiation are substantial and have prompted the search for a more biologically driven therapeutic strategy, e.g., targeting receptor tyrosine kinases (RTKs) differentially expressed between tumors and normal tissues 3–5.

Among the RTKs, the epidermal growth factor receptor (EGFR) has emerged as an independent prognostic factor for several cancers, including head and neck squamous cell carcinoma (HNSCC), and an appealing target for therapeutic intervention 6,7. Cetuximab, a monoclonal antibody against EGFR, was developed as a novel, less toxic, frontline therapy when combined with radiation therapy (RT) for patients with locally advanced HNSCC 7,8. This therapy was found to increase LR control and OS rates by 10–15%, a similar magnitude as that achievable with combinations of radiation with cytotoxic chemotherapy. However, despite this observed therapeutic benefit, greater than 50% of patients still experienced LR relapse 9. Thus, a substantial group of patients appears to be resistant to the radiosensitization derived by EGFR inhibition.

Emerging data show that there is crosstalk between EGFR and other members of the EGFR family with the insulin-like growth factor receptor (IGF-1R) pathway 10,11. In addition, many neoplasms, including HNSCCs and non-small cell lung cancers (NSCLCs) express high levels of IGF-1R and such overexpression is associated with resistance to therapy 11,12. Thus, it is logical to hypothesize that cotargeting EGFR and IGF-1R pathways will overcome the cellular resistance to RT. Here we assessed whether the combination of two therapeutic monoclonal antibodies, cetuximab, and IMC-A12 that specifically target EGFR and IGF-1R, respectively, would enhance HNSCC tumor response to radiation.

## Materials and Methods

### Cell culture

HNSCCs: FaDu and Detroit-562 were from American Type Culture Collection (Manassas, VA); UMSCC-1 and UMSCC-22A were from Dr. Thomas E. Carey, University of Michigan; HN-5 was from Dr. D. M. Easty, Ludwig Institute for Cancer Research, London; MDA-183 (established by Dr. Peter G. Sacks, New York University) and SqCC/Y1 were from Dr. Jeffrey Myers, the University of Texas M.D. Anderson Cancer Center (UTMDACC). All cell lines were maintained in appropriate culture media supplemented with 10% fetal calf serum and 10,000 U/mL of penicillin-streptomycin. Short tandem repeat profiling cell line validation was performed by the Characterized Cell Line core at UTMDACC (supported by CA016672).

### Drugs

Cetuximab (Erbitux) and cixutumumab (IMC-A12) were obtained from Imclone Systems Inc., Somerville, NJ.

### Cell viability assay

The cells were plated in a 96-well plate and treated the next day with various concentrations (15–120 nmol/L) of cetuximab, and cell viability assay was performed as described 13.

### Immunoprecipitation and Western blot analysis

Cells lysates were subjected to immuno-precipitation (IP) and Western blot analysis as described 13. Primary antibodies were bought from Cell Signaling Technology Inc. (Beverly, MA) (EGFR, IGF-1R, Akt, MAPK) and from Chemicon, Inc. (Pittsburgh, PA) (*β*-actin, *α*-tubulin). Protein A Sepharose beads were from GE Healthcare Biosciences (Uppsala, Sweden). Secondary antibodies were from Amersham Biosciences (Arlington Heights, IL). Immunoreactions were visualized using ECL-Plus detection system from Amersham Biosciences (Arlington Heights, IL) and analyzed by Typhoon scanner with ImageQuant software from Molecular Dynamics Inc. (Sunnyvale, CA).

### Clonogenic cell survival determination

Known number of cells were plated in triplicate and exposed to cetuximab (30 nmol/L), IMC-A12 (100 nmol/L) or vehicle for 6 h and then irradiated with graded doses (2, 4, or 6 Gy) of *γ*-rays using a ^137^Cs source (3.7 Gy/min). The cells were left in the incubator with the drug in the medium and the medium was changed 66 h after radiation. The survival curves were constructed by fitting the average survival levels using least squares regression by the linear quadratic model 14 after normalizing for the cytotoxicity induced by the drug(s) alone.

### Immunocytochemistry (ICC) for γ-H2AX and 53BP1 foci

Cells were grown on cover-slips and at specified time points after exposure to cetuximab, 4 Gy of radiation, or both, cells were fixed and processed for immunofluorescence. Foci were visualized using Leika Microsystems (Wetzlar, Germany) and analyzed using SPOT software (SPOT Imaging solutions, Diagnostic Instruments, Sterling Heights, MI).

### In vivo tumor growth delay assay

Solitary tumors were generated by inoculating 1 × 10^6^ cells into the right hind leg of mice 15. Treatments were initiated when tumors grew to 7 or 8-mm diameter (designated as day 0). Control mice received no treatment. Local tumor RT was delivered (5 Gy/min) using a small animal ^137^Cs irradiator. Fractions of 2 Gy were given twice a day with the first dose delivered on day 0. Cetuximab or IMC-A12 was administered (i.p.) at a dosage of 1 mg per mouse given for 3 (Detroit-562) or 4 (FaDu) times at 3-day intervals from day 0. When combined with RT cetuximab or IMC-A12 was given 2 h before RT. When both cetuximab and IMC-A12 were administered concurrently, IMC-A12 was administered 2 h before cetuximab. Regression and regrowth of tumors were expressed as the time in days for tumors in the treated groups to grow from 7 or 8-mm to 12-mm in diameter.

### Statistical analysis

Results presented as the mean±standard error were from at least three independent experiments. Values of survival fractions were tested for statistical significance by Student's *t-*test and *P* < 0.05 were considered statistically significant.

## Results

### Differential effect of cetuximab on cell viability

**Figure 1:**
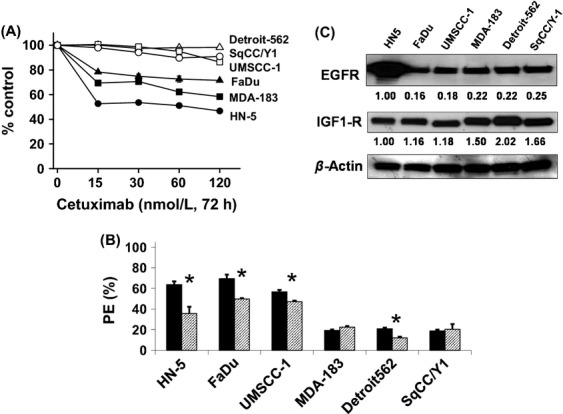
(A) Effect of cetuximab on survival of cells in culture. Cells were exposed to various doses of cetuximab (15–120 nmol/L) for 72 h and MTT assay was performed. Data shown are means ± SE from three independent experiments. (B) Effect of cetuximab on the colony forming ability or plating efficiency (PE) of cells assessed by clonogenic cell survival assay, in the absence (black bars) or presence (striped bars) of cetuximab (30 nmol/L, 72 h). Data shown are the means±SE of three independent experiments. *P ≤ 0.05. (C) Western blots showing the expression levels of EGFR and IGF1R in six HNSCC lines. Numbers shown below protein bands are relative intensities of the bands with the level in HN-5 cells as 1.0. Western blots shown are representative of three independent experiments.

First, dose-dependent cytotoxicity of cetuximab alone was determined both by MTT assay and clonogenic cell survival assay in six HNSCC lines. As shown in Figure1A, HN-5 was relatively more sensitive than the other lines. A 15 nmol/L dose of cetuximab reduced the cell viability to 50–80% depending on the cell type in HN-5, FaDu, and MDA-183. However, three of the six cell lines (Detroit-562, UMSCC-1 and SqCC/Y1) tested did not respond to cetuximab-induced toxicity within the 72 h of exposure to cetuximab. In clonogenic cell survival assays HN-5, FaDu, Detroit-562, and UMSCC-1 cells showed reduced number of colonies when exposed to 30 nmol/L cetuximab (*P =* *0.05). Cetuximab did not have any effect on MDA-183 and SqCC/Y1 (Fig.1B).

### Levels of EGFR and IGF1R expressed in HNSCC lines

To determine whether the expression levels of EGFR in these cell lines correlate with their response to cetuximab, Western blot analysis was performed. With the exception of HN-5, EGFR expression did not correlate with the response to cetuximab (Fig.1C). Since cross-talk between EGFR and IGF-1R exists we tested the expression level of IGF-1R in these lines. All cell lines examined expressed IGF-1R (Fig.1C).

### Differential response of cells to combined exposure to cetuximab and radiation

**Figure 2:**
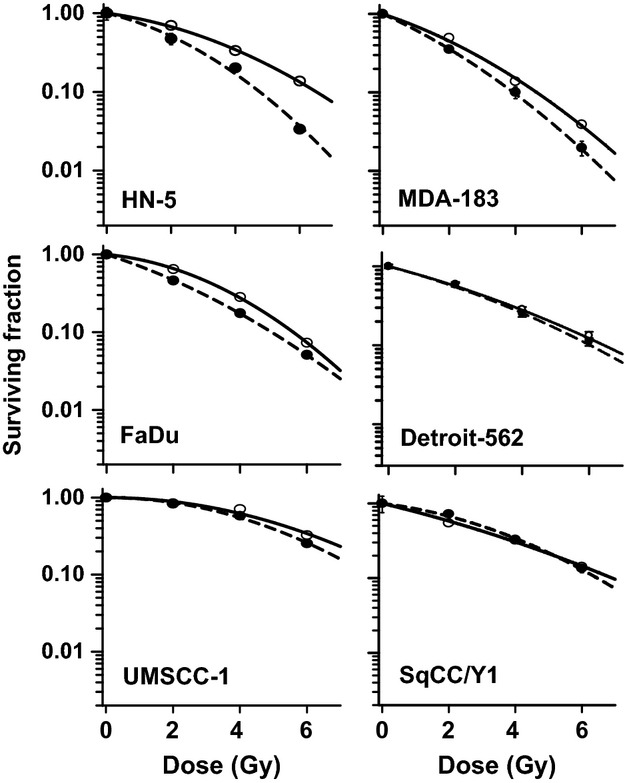
Effect of cetuximab on radiosensitivity of HNSCC lines in culture. Cells were treated with cetuximab as described under Methods. Data shown are means±SE from three independent experiments.

When combined with radiation, cetuximab enhanced the radiosensitivity of only HN-5 cells. A dose enhancement ratio (DER) at survival fraction level of 0.5 was 1.62 for HN-5 cells (Fig.2). Cetuximab had a lesser radiosensitizing effect on MDA-183 and FaDu cells with the DER of 1.17 and 1.31, respectively and no radiosensitizing effects on the remaining cell lines.

### Persistence of radiation-induced nuclear γ-H2AX and 53BP1 foci

**Figure 3:**
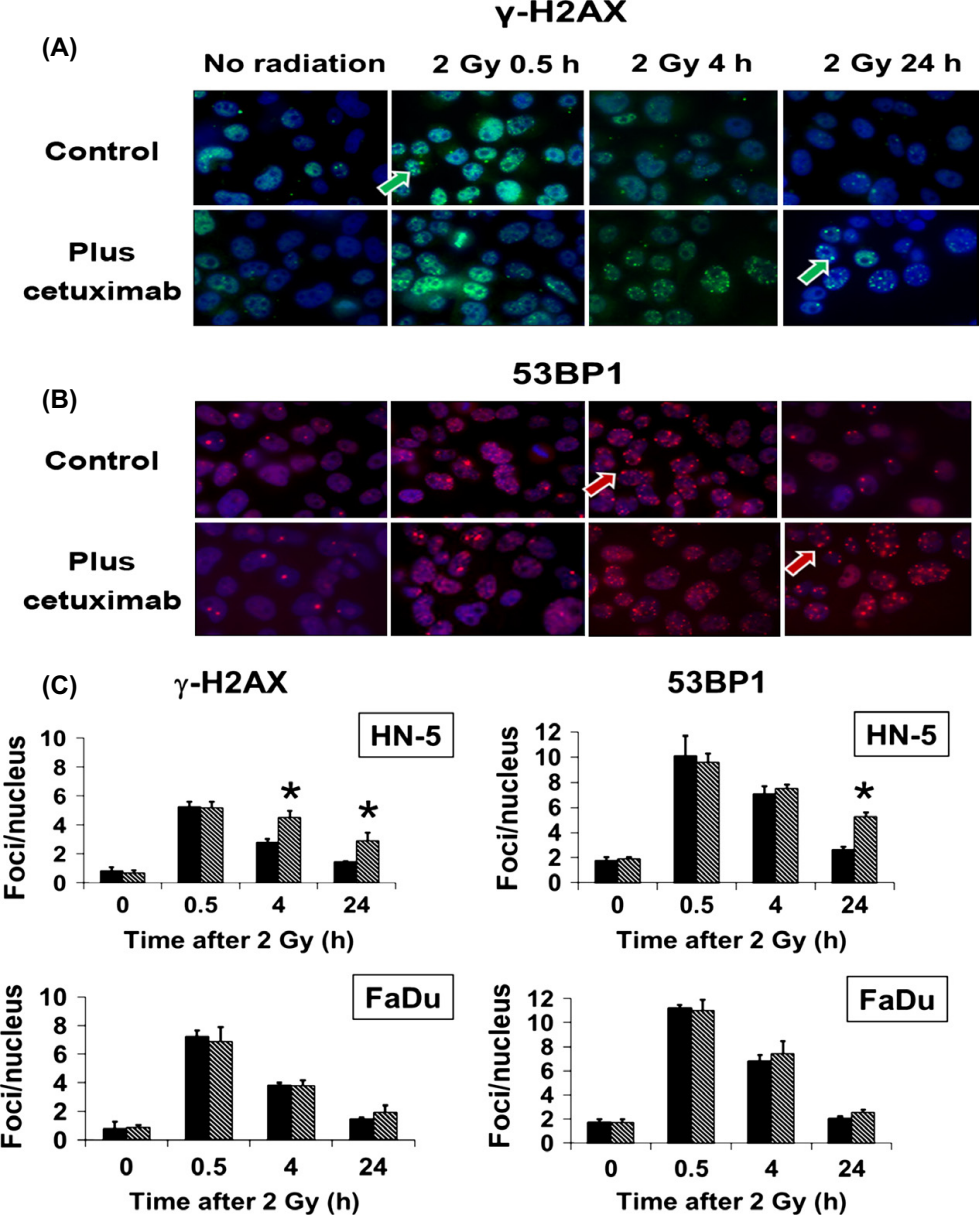
Effect of cetuximab, radiation, or both on nuclear *γ*-H2AX and 53BP1 foci in HN-5 cells. Immunocytochemistry (ICC) with *γ*-H2AX or 53BP1 antibody was performed as described in Methods section. (A) Shown are representative nuclei of HN-5 cells with *γ*-H2AX foci (FITC stain, green) after specified treatments. Green arrows: *γ*-H2AX foci. (B) Shown are representative nuclei of HN-5 cells with 53BP1 foci (Cy-3 stain, red) after specified treatments. Red arrows: 53BP1 foci. (C) Quantitative analysis of *γ*-H2AX and 53BP1 foci in HN-5 and FaDu cells. Foci numbers were counted and plotted as average number of foci per nucleus against time after irradiation. Data shown are means±SE of more than 100 nuclei from two independent experiments. *P ≤ 0.05.

To determine whether resistance to cetuximab-mediated radiosensitization was associated with alterations in DNA repair processes we assessed the effect of cetuximab on the kinetics of repair of radiation-induced DNA double-stand breaks (DSBs) in HN-5 and FaDu cells. As shown in Figure3A and B, 2 Gy of radiation induced *γ*-H2AX and 53BP1 foci formation in >90% of cells by 30 min. A rapid reduction in the number of foci was observed in both cell lines by 4 h and reaching close to the basal level by 24 h. Addition of cetuximab extended the presence of these foci at 4 and 24 h in HN-5 cells but not in FaDu cells (Fig.3C). These results suggest that in HN-5 cells the increase in radiosensitivity due to cetuximab is related suppression of DNA repair processes.

### The effects of cetuximab inhibition on EGFR and IGF-1R expression

**Figure 4:**
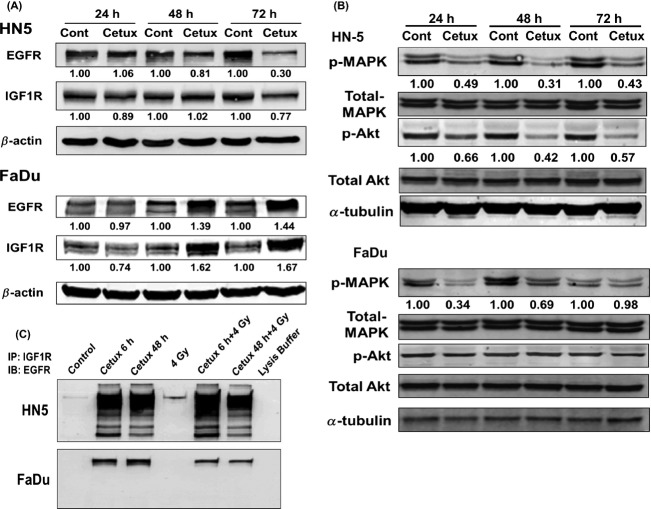
(A) Effect of cetuximab on EGFR and IGF-1R expression levels. Cells were exposed to cetuximab and subjected to Western blot analysis. Numbers shown below protein bands are relative intensities with levels in untreated control cells as 1.0. Western blots shown are representative of two independent experiments. (B) Effect of cetuximab on p-MAPK and p-Akt expression levels. Cells were exposed to cetuximab and subjected to Western blot analysis. Numbers shown below protein bands are relative intensities with levels in untreated control cells as 1.0. Western blots shown are representative of two independent experiments. (C) Effect of cetuximab, radiation, or both on dimerization of EGFR and IGF1R. Cells were exposed to either cetuximab and/or 4 Gy and collected 10 min after irradiation. Whole cell lysates were subjected to immunoprecipitation (IP) with IGF-1R antibody and immunoblotted (IB) with EGFR antibody. Shown are representative Western blots of two independent experiments. Cont: Untreated control; cetux: cetuximab. p-MAPK: phosphorylated form of MAPK; p-AKT: phosphorylated form of Akt.

Because EGFR and IGF-1R participate in significant cross-talk, we examined the effects of EGFR inhibition on IGF-1R expression. As shown in Figure4A, in HN-5 cells, (cetuximab-sensitive) treatment with cetuximab led to a reduction in EGFR level. Interestingly, IGF-1R level was also reduced. Conversely, in FaDu, exposure to cetuximab induced EGFR expression as well as IGF-1R expression levels. These data led us to hypothesize that in FaDu cells IGF-1R was in part mediating resistance to cetuximab-induced radiosensitization via dimerization with EGFR. We tested changes in two downstream signaling responses after exposing the cells to cetuximab. As shown in Figure4B, cetuximab suppressed the expression of phosphorylated form of mitogen activated protein kinase (p-MAPK) in both HN-5 and FaDu cells. However, phosphorylated form of Akt was suppressed only in HN-5 cells and not in FaDu cells.

### Induction of dimerization of EGFR and IGF-1R by cetuximab

To determine whether exposure to cetuximab and RT induced cross-talk between EGFR and IGF-1R, we performed coimmunoprecipitation. As shown in Figure4C, exposure of cells to cetuximab-induced dimerization of EGFR with IGF-1R. Though there was an apparent increase in binding of EGFR and IGF-1R 10 min after 4 Gy, it was relatively a small event when compared to the effect of cetuximab. Additionally, in HN-5, multiple components of EGFR were evident possibly suggesting ubiquitination and subsequent degradation of EGFR. However, there were no such multiple protein bands in FaDu cells. Appearance of such low molecular weight components of EGFR correlated with the observed radiosensitizing effect of cetuximab in HN-5 cells.

### Differential response of cells to combined exposure to cetuximab, IMC-A12, and radiation

**Figure 5:**
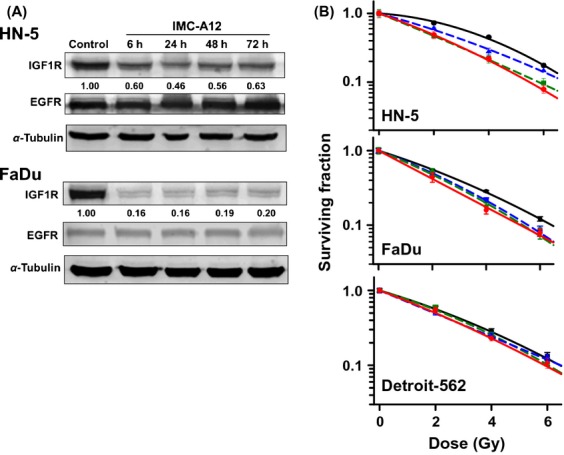
(A) Effect of IMC-A12 on IGF-1R and EGFR expression levels. Cells were exposed to IMC-A12and subjected to Western blot analysis. Numbers shown below protein bands are relative intensities with levels in untreated control cells as 1.0. Western blots shown are representative of two independent experiments. (B). Effect of cetuximab and IMC-A12 on radiosensitivity of HNSCC lines in culture. Cells were treated with cetuximab and/or IMC-A12 and exposed to radiation as described under Methods. Survival curves were constructed with normalized values for the cytotoxicity induced by cetuximab/IMC-A12. Data shown are means ± SE from three independent experiments. RT only: black solid line; cetuximab + RT: green dash line; IMC-A12 + RT: blue dash line; cetuximab + IMC-A12 + RT: Red solid line.

First we tested the effect of IMC-A12 on the levels of IGF-1R and EGFR. IMC-A12 alone decreased the levels of IGF-1R in HN-5 cells by about 40% and in FaDu cells the decrease was about 80%. IMC-A12 had no measurable effect on EGFR expression (Fig.5A). We then determined clonogenic survival levels after exposing cells to cetuximab and IMC-A12 or the combination and RT. In HN-5 cells (sensitive to cetuximab-induced radiosensitivity), IMC-A12 as a single agent enhanced the cell radiosensitivity. However, when combined with cetuximab, IMC-A12 did not influence the cell radiosensitivity beyond the effect of cetuximab (Fig.5B). In FaDu cells, IMC-A12 enhanced the radiosensitivity of cells moderately, similar to the effect of cetuximab and when combined with cetuximab there was no significant increase in cell radiosensitivity. In Detroit-562 cells, these two agents had no effect on cell radiosensitivity.

### Effect of cetuximab, IMC-A12, and radiation on the growth of tumor xenografts generated in mice

**Figure 6:**
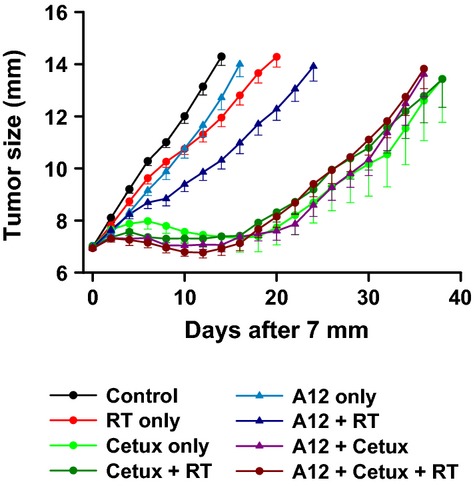
Antitumor efficacy of cetuximab and IMC-A12 with fractionated radiation: Tumor growth delay assay. FaDu tumor xenografts were generated in mice and treated with cetuximab (Cetux) and/or IMC-A12 (A12) along with fractionated doses of radiation (RT) as described under Methods section. Each data point represents the mean size of 8 tumors ± SE.

To determine the efficacy of these two agents in enhancing radiosensitivity under in vivo settings, FaDu tumor xenografts were generated in mice and the treatments were initiated when the tumor xenografts reached 7-mm in size. As shown in Figure6, fractionated doses of RT slightly delayed the tumor growth when compared to the untreated tumors. Cetuximab as a single agent dramatically suppressed the tumor growth. However, when RT and cetuximab were combined, there was no further increase in tumor growth delay (TGD). Whereas IMC-A12 as a single agent as well as with RT slowed down the tumor growth considerably, addition of IMC-A12 to cetuximab and RT did not further delay the tumor growth. Table1 summarizes the TGD data presenting the number of days taken for tumors in each group to reach from 7-mm to 12-mm in size.

**Table 1:** FaDu tumor growth delay assay.

Treatments	Mean number of days ± SE
Control	10.1 ± 0.5
RT	14.2 ± 0.9
Cetuximab	43.1 ± 7.4^1^
Cetuximab + RT	34.4 ± 2.6
IMC-A12	12.8 ± 0.8
RT + IMC-A12	19.3 ± 1.3
Cetuximab + IMC-A12	33.8 ± 1.7
RT + Cetuximab + IMC-A12	32.8 ± 2.2

Effects of radiation (RT), cetuximab, IMC-A12, or combinations of these three agents on the tumor growth. Mean number of days for the tumors to reach 12 mm in size.

1 Control vs. Cetuximab: *P *≤ 0.5

**Figure 7:**
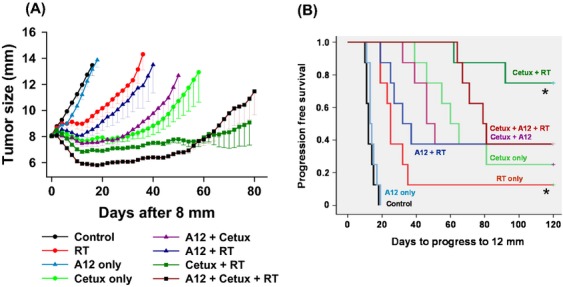
Antitumor efficacy of cetuximab and IMC-A12 with fractionated radiation. (A) Tumor growth delay assay. Detroit-562 tumor xenografts were generated in mice and treated with cetuximab (Cetux) and/or IMC-A12 (A12) along with fractionated doses of radiation (RT) as described under Methods section. Each data point represents the mean size of eight tumors ± SE. (B) Tumor growth delay assay data analyzed by the Kaplan–Meier method. The progression-free survival against the number of days to reach 12-mm in size is plotted. The survival curves were generated after treatments with radiation only (RT), and in combination with cetuximab (Cetux), or IMC-A12 (A12), or both as described above. *P ≤ 0.05.

Similar TGD assay was performed in Detroit-562 tumor xenografts. Treatments were initiated when the tumor xenografts reached 8 mm in size. As shown in Figure7A, fractionated doses of RT delayed the tumor growth markedly when compared to the untreated control group. Whereas cetuximab given as a single agent delayed the tumor growth significantly, IMC-A12 alone did not have any significant effect. The combination of RT and cetuximab had a dramatic effect in delaying the tumor growth and six out of eight mice tumors were reduced to nonmeasurable size that were considered as cured. The addition of IMC-A12 to RT had a minimal additional effect on tumor growth compared to RT alone. When these two agents were applied concurrently along with RT, initially, there was a dramatic suppression of tumor growth that was more than that of combination of RT with cetuximab or IMC-A12; however, by day 50, this effect was lost and the converse was observed. In this triple therapy group, there were three out of eight tumors reached cured levels. Table2 shows the average number of days for tumors in each group to grow from 8-mm to 12-mm in diameter. In Figure6B, the progression-free survival against the number of days to reach 12-mm in size is plotted analyzed by Kaplan–Meier method. The cetuximab plus RT group showed the best response as around 120 days in to the experiment, six out of eight mice exhibited progression-free survival compared to three out of eight mice in triple therapy group.

**Table 2:** Detroit-562 tumor growth delay assay.

Treatments	Mean number of days ± SE
Control	13.4 ± 1.3
RT	25.9 ± 2.1^1^
Cetuximab	52.4 ± 4.5^2^
Cetuximab + RT	77.3 ± 15.7^3^
IMC-A12	13.7 ± 0.7
RT + IMC-A12	28.6 ± 3.1
Cetuximab + IMC-A12	42.8 ± 3.4
RT + Cetuximab + IMC-A12	73.1 ± 3.6

Effect of radiation (RT), cetuximab, IMC-A12, or combinations of these three agents on the tumor growth. Mean number of days for the tumors to reach 12 mm in size.

1 Control vs. RT; ^2^Control vs. Cetuximab; ^3^RT vs. Cetuximab + RT: *P* ≤ 0.5.

## Discussion

Because activation of IGF-1R-mediated signaling has been associated with cancer cell resistance to anti-EGFR therapy and also to RT 16,17, cotargeting EGFR and IGF-1R pathways in conjunctions with RT is expected to yield a better outcome in controlling tumor growth. In addition, anti-IGF-1R therapy was shown to augment the response of certain malignant tumors to RT in preclinical settings 18,19. It has been reported that RT activates both EGFR and IGF-1R signaling 20–22 and inhibition of EGFR and IGF-1R activation resulted in overcoming the resistance to RT 23. Inhibition of EGFR and IGF-1R pathways using their respective therapeutic antibodies presented promising results in controlling tumor growth in several preclinical models 24,25. We observed that in vitro response to either cetuximab or IMC-A12 when combined with RT depended on the cell line. In HN-5 cells, which are sensitive to the radiosensitizing effects of cetuximab, inhibition of DNA DSB repair seemed to be the underlying mechanism of this effect. These data are consistent with our previous report that cetuximab enhanced the cell radiosensitivity by blocking the repair of RT-induced DSBs 26. When IMC-A12 was added concurrently to the cetuximab plus RT regimen in vitro*,* none of the cell lines exhibited any increase in radiosensitivity suggesting that molecular components other than IGF-1R may have provided survival advantage for these cells.

Binding of EGFR and IGF-1R has been reported previously 27. However, the functional outcome of this interaction has not been understood. Our data showed that in HN-5 and FaDu cells, cetuximab-induced binding of EGFR and IGF-1R was apparent in IP and immunoblot analyses. It has to be noted that in HN-5 cells and not in FaDu cells there was an indication that EGFR was degraded. These data are consistent with the earlier reports on the effect of cetuximab leading to internalization and degradation of EGFR 28. Additionally, cetuximab suppressed the expression of p-Akt only in HN-5 cells suggesting that the survival pathway was inhibited in HN-5 and not in FaDu cells. Taken together, our in vitro data suggest that HN-5, which expresses high levels of EGFR, showed an increase in radiosensitivity in response to EGFR inhibition and additional inhibition of IGF-1R did not further enhance the radiosensitivity. Interaction of EGFR and IGF-1R has been described to be mediated by the ligands of these two receptors or by other receptors and downstream effector proteins 29,30. Though existence of strong interaction between these two receptors is well established it is unclear how the interaction between these two receptors could alter the cellular response to RT. Our data showed no correlation between the binding of these two receptors and cell radiosensitivity.

To investigate these findings further, in vivo studies were performed using FaDu and Detroit-562 tumor xenografts. Contrary to our in vitro data, in Detroit-562, the cetuximab plus RT group as well as the triple therapy group (cetuximab + IMC-A12 and RT) showed marked overall TGD and tumor regression in six out of eight mice and three out of eight mice, respectively. Taken together these data showed that cetuximab plus RT regimen appear to yield a better outcome than the triple therapy regimen in Detroit-562. Additionally, since the cetuximab and IMC-A12 treatments were limited to only three times at 3-d intervals, differential up-regulation of EGFR or IGF-1R after the termination of treatments may have contributed to accelerated tumor growth. Thus, prolonged exposure to these agents may have been beneficial in controlling tumor growth. These findings confirm the importance of maintenance therapy consistent with our previous report 31.

Previously, we have reported that inhibition of these two pathways using panitumumab (anti-EGFR antibody) and ganitumab (anti IGF-1R antibody) enhanced the FaDu tumor response to radiation 32. Panitumumab as a single agent as well as in combination with RT evoked a moderate delay in FaDu tumor growth. In contrast, cetuximab as a single agent suppressed FaDu tumor growth profoundly. Such a difference in FaDu tumor response to panitumumab and cetuximab may be due to the difference in the binding characteristics of these therapeutic antibodies to EGFR. Cetuximab is a chimeric (mouse/human) monoclonal antibody. Panitumumab is a humanized monoclonal antibody. Humanized antibodies are distinct from chimeric antibodies; the latter also have protein sequences that are more similar to human antibodies, but carry a larger stretch of nonhuman protein. Thus, due to these differences the response of FaDu tumor xenografts may be different. Additionally, in the current study adding IMC-A12 to cetuximab and RT treatment regimen did not have any effect on FaDu tumor growth, which is consistent with our in vitro data.

In conclusion, though cetuximab or IMC-A12 individually has the potential of enhancing tumor response to RT, concurrent application of these two agents did not yield additional benefit in suppressing the growth of two HNSCC tumor models tested in vivo. These data suggest that RTKs other than EGFR and IGF-1R and/or potential downstream effector proteins might compensate for the loss of EGFR and IGF-1R activity. Identification of specific compensatory pathways and targeting them will yield a better therapeutic outcome.
